# Study of Properties and Characteristics of a Foam Glass from a Mixture of Glass Shards and Perlite

**DOI:** 10.3390/ma18184422

**Published:** 2025-09-22

**Authors:** Ilja Horonko, Pavels Tihomirovs, Aleksandrs Korjakins

**Affiliations:** Institute of Sustainable Building Materials and Engineering Systems, Faculty of Civil and Mechanical Engineering, Riga Technical University, Kipsalas Str. 6A, LV-1048 Riga, Latvia; ilja.horonko@edu.rtu.lv (I.H.); pavels.tihomirovs@edu.rtu.lv (P.T.)

**Keywords:** foamed glass, ground perlite, waste glass recycling, thermal insulation, material porosity, thermal treatment, thermal conductivity

## Abstract

The current study presents the development and optimisation of foam glass manufactured from recycled glass shards and expanded ground perlite, targeting enhanced structural and thermal performance for sustainable building applications. By investigating various particle size fractions (“125 μm”, “250 μm”, “500 μm”) and sintering temperatures (800–850 °C), we achieved a foam glass with superior compressive strength and uniform porosity. Notably, samples utilising a homogeneous 500 μm particle fraction sintered at 850 °C exhibited the highest compressive strength of 2.17 MPa, coupled with open porosity uniformity and stable structural matrix formation. Density values in this fraction decreased from 321 to 263 kg/m^3^, indicating effective foaming and well-developed open porosity that balances mechanical integrity and thermal insulation. The optimised thermal regime minimised crystalline phase formation, preserving low thermal conductivity and mechanical stability. Compared to heterogeneous composites, the homogeneous fractions demonstrated significantly improved strength-to-porosity ratios, ensuring predictable mechanical performance and competitive thermal insulation properties. These findings underline the material’s potential as a cost-effective, environmentally friendly insulation solution that meets or exceeds existing standards, with promising applications in energy-efficient construction.

## 1. Introduction

In the modern construction industry, energy efficiency has become a primary strategic priority, driven by both economic and environmental concerns. One of the most critical factors affecting a building’s energy performance is high-quality thermal insulation. Good insulation not only reduces energy loss but also improves indoor comfort and lowers operating costs. Today’s thermal insulation materials vary widely, and selection depends on specific requirements such as thermal conductivity, moisture resistance, fire safety, mechanical strength, and durability. The European market for thermal insulation is largely dominated by mineral wool and plastic foams (such as EPS and XPS), whose popularity stems from their availability and well-established manufacturing technologies [1,2,3,4,5].

However, these materials also have drawbacks, including relatively short service lives in humid environments, flammability, and negative environmental impacts during both production and disposal. The holistic environmental impacts of insulation materials were assessed using life cycle assessment (LCA). Three major insulation materials were evaluated: wool-based materials (mineral wool or glass wool), expanded polystyrene (EPS), and polyurethane foam [6]. The analysis used a cradle-to-grave scope and focused on five representative environmental indicators: global warming potential (100-year horizon), acidification potential, eutrophication potential, ozone depletion potential, and human toxicity potential. The assessment was performed using GaBi LCA software, GaBi Version 9.5.2.49, GaBi Database Edition 2020, CUP 2020.02. The results showed that polyurethane foam had the greatest impact, especially for global warming, eutrophication, and human toxicity. Expanded polystyrene and wool-based materials performed better than polyurethane foam in most categories. For example, wool-based insulation was found to produce 2.1 × 10^4^ kg CO_2_-eq (GWP) and 760.1 kg DCB-eq (HTP), while expanded polystyrene showed similar GWP, AP, and EP values of 2.1 × 10^4^ kg CO_2_-eq, 23.3 kg SO_2_-eq, and 2.7 kg PO_4_-eq, respectively [6].

Alternative thermal insulation solutions such as foam glass are gaining importance due to their high fire resistance, moisture resistance, chemical stability, and long service life. Foam glass is primarily produced from recycled glass waste, making it an environmentally friendly alternative to traditional materials. Moreover, foam glass is completely non-combustible, contains no organic substances, is non-toxic, and retains its thermal insulation properties even under extreme conditions.

Although foam glass offers many advantages, its wider adoption is currently limited by high production costs. In 2023 the foam glass market was valued at USD 2.11 billion. Forecasts indicate it will grow from USD 2.22 billion in 2024 to USD 3.14 billion by 2031, representing a compound annual growth rate (CAGR) of 7.50% from 2024 to 2031. The primary factors driving this market expansion are increasing construction activity and growing demand for materials with the unique properties of foam glass.

The increasing cost of foam glass, such as FOAMGLAS (manufacturer Owens Corning, London, United Kingdom), is related to several factors, including its production features and the material’s unique properties. The production of foam glass requires melting cullet at high temperatures (approximately 1000 °C), resulting in high energy consumption. This complex process also involves the addition of foaming agents, which necessitates the use of specialised equipment and technologies.

Therefore, it can be concluded that foam glass possesses a wide range of advantages—it can serve both as a thermal and acoustic insulating material, as well as a load-bearing material. Thus, the application of new developed glass foam insulation material meets several challenges formulated in the EU Green Deal aims [7]. They are as follows: decreasing consumption of energy for heating and cooling the buildings by applying high-quality insulation materials, decreasing energy consumption for producing sustainable insulation material, and reducing the amount of industrial and municipal waste. The main drawback currently limiting its use is the high cost of the final product. This leads to the logical conclusion that there is a need to develop an economically and environmentally advantageous foam glass formulation that reduces the overall costs of the final product. According to research [8] sintering temperature for glass foam is in the range 600–900 °C. The sintering temperature for glass foam used in civil engineering reaches 920–1050 °C [9,10]. More specifically, a composition must be achieved in which the foaming temperature is reduced, preferably below 850 °C. At the same time, insulation and strength characteristics must not significantly deteriorate. It is also necessary to incorporate more readily available materials into the mixture to further reduce the overall cost of the final product.

Foam glass produced from a mixture of glass shards and perlite represents a sustainable approach to recycling waste glass while incorporating the unique properties of perlite, a naturally occurring expanded volcanic glass. The synthesis of such foam glass materials involves mixing finely crushed glass with a controlled amount of perlite, followed by a sintering process at temperatures sufficient to soften the glass while preserving the pore structure introduced by the perlite.

During the heat treatment, the softened glass acts as a binding matrix that encapsulates the perlite particles. Perlite, which inherently possesses a low bulk density and an open porous structure, functions as both a foaming agent and a lightweight aggregate. This dual role promotes the formation of a cellular structure that imparts high thermal insulation and sound absorption properties to the final foam glass product. The contribution of perlite is particularly critical in enhancing the uniformity and stability of the pore network, which influences the mechanical strength and thermal conductivity of the composite material.

The primary objective of this study is to develop optimal production methods for foam glass that enhance thermal insulation and mechanical properties. Based on theoretical and experimental research, a new methodology for foam glass production using secondary materials (perlite) was developed. The approach involves systematic modifications to the glass–perlite mixture and processing conditions, including the following:

- Varying the glass-to-perlite ratio to balance porosity and load-bearing wall fraction (higher perlite content tends to increase total porosity and lower thermal conductivity, while more glass promotes stronger pore walls and higher compressive strength).
- Controlling perlite particle size and distribution to influence pore nucleation and pore-size distribution (finer particles favour more uniform, smaller pores and lower thermal conductivity, whereas coarser particles can increase open porosity and reduce.

## 2. Materials and Methods

### 2.1. Glass

One of the main components used in foam glass production is glass. In this study, various types of waste glass containers were used as raw materials, including both transparent and coloured glass. To increase the efficiency of the processing procedure and reduce the costs of raw material pre-treatment, the glass containers were not sorted by colour.

The chemical composition of recycled glass containers depends on several factors, including the type of glass, the manufacturer, the colour additive, and the conditions of use. However, for the majority of glass container types used in food production and other industries, the primary component is Sodium Calcium Silicates. The approximate chemical composition of traditional glass used for the production of bottles and other containers from the municipal waste is presented in Table 1:

Coloured glass waste, in addition to the main components, may contain other oxides such as chromium oxide (Cr_2_O_3_), which imparts a green tint, or cobalt oxide (CoO), which produces a blue hue. Although present in low concentrations, these components can influence the physical and chemical properties of the final product, foam glass.

### 2.2. Foaming Agent

In this experiment, limestone (calcium carbonate, CaCO_3_) was selected as the foaming agent. Limestone was chosen due to its availability, stable chemical composition, and its ability to decompose upon heating, which promotes the formation of a porous structure within the glass matrix [11,12,13,14,15,16,17].

Limestone or calcium carbonate decomposes when heated to high temperatures, releasing carbon dioxide (CO_2_) and forming calcium oxide (CaO) according to the following chemical reaction:

CaCO_3_→CaO + CO_2_**↑**

This process begins at a temperature of approximately 700–800 °C [18]. The pore size, uniformity, and density depend on the decomposition temperature and rate of calcium carbonate, as well as on the viscosity of the glass melt at different stages of heating.

The particle size of limestone influences the foaming process. Finely ground limestone decomposes more rapidly and uniformly, resulting in the formation of small, evenly distributed pores. Larger particles may decompose more slowly, leading to irregular porosity and the formation of structural defects in the foam glass.

### 2.3. Perlite

Perlite is a natural volcanic material that expands upon heating, forming a porous structure. In this experiment, crystallised perlite in granular form is used, which is pre-ground into a powder and further incorporated as part of the raw material mixture. The perlite was received from the Department store. Greece is the country of origin. The Chemical composition of the perlite is presented in Table 2.

Alaa M. Rashad considered the influence of perlite embedded in the different materials as aggregate on the thermal insulation properties and mechanical properties of these materials [19]. The perlite inclusion in the foam glass may result in a positive effect of decreasing thermal conductivity with insignificant strength decrease in glass foam.

Perlite possesses high thermal stability and can withstand the temperatures required for glass foaming (approximately 850–1050 °C). This property allows its inclusion in the batch without compromising the structural integrity of the material at elevated temperatures.

The porous structure of perlite facilitates the formation of lightweight and porous materials. The aim of adding perlite to the foam glass composition is to enhance thermal insulation properties and reduce the final density of the product

To ensure the successful use of perlite in foam glass production, its granular form must first be ground into a fine powder. This process is essential to ensure the uniform distribution of perlite within the glass matrix and to prevent the formation of large structural defects in the foam glass.

### 2.4. Alkaline Component

As part of the experiment to produce foam glass, sodium hydroxide (NaOH) was introduced into the raw material mixture. Its use served multiple purposes: to reduce the viscosity of the glass melt, thereby facilitating the foaming process at lower temperatures, and to act as an auxiliary foaming agent. Sodium hydroxide (NaOH) technical grade was applied in the investigation.

The addition of NaOH lowers the melting temperature of the glass batch, enabling foaming at temperatures below those required by conventional technologies [20].

In addition to its primary function—reducing the viscosity of the glass melt—sodium hydroxide also acts as a weak foaming agent, improving the wettability of perlite and thereby enhancing its adhesion to other components of the mixture.

The quantity of sodium hydroxide in the mixture must be carefully controlled. Excessive NaOH content can cause an excessive reduction in the viscosity of the glass melt, making it too fluid to retain gas bubbles, as well as degrading the mechanical properties of the foam glass. The optimal concentration of NaOH is generally in the range of 5–10% of the total weight of the raw materials [17].

A key consideration when adding NaOH is its potential to increase the tendency of the glass matrix to crystallise during the cooling phase, which may deteriorate the physical properties of the foam glass if the crystallisation process is not properly controlled. Crystallisation within the foam glass structure can impair mechanical performance, as crystalline regions tend to be more brittle compared to the amorphous phase. The amorphous structure of foam glass ensures favourable thermal insulation properties, whereas a crystalline structure has an adverse effect on these characteristics.

### 2.5. Distilled Water

In the foam glass formation experiment, distilled water was selected as one of the auxiliary materials. Its use was essential to perform the function of a binding agent during the preparation of specimens from the raw material mixture.

Distilled water was added to moisten the dry components of the mixture, ensuring their uniform blending and the formation of a homogeneous mass. This facilitated the moulding of specimens without the occurrence of cracks or defects during the initial processing stage.

Distilled water was explicitly employed to eliminate the risk of introducing foreign substances that could be present in regular tap water.

### 2.6. Initial Components Grinding

The glass crushing process for subsequent foam glass production plays a crucial role, as it enables the achievement of the desired particle size for effective foaming and production of a final material with the required properties [21].

**Figure 1:**
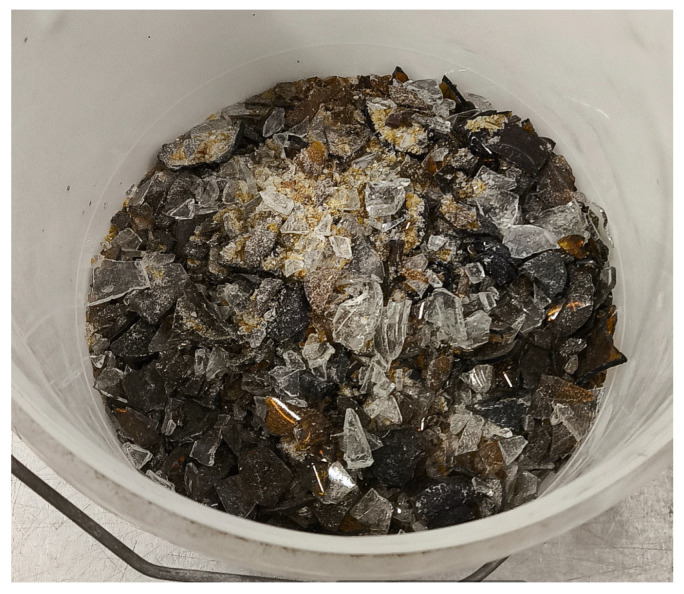
Glass fragments after manual crushing using a sledgehammer.

In the initial stage, glass containers were mechanically broken—manually using a sledgehammer (Figure 1). This approach allowed for the acquisition of large fragments (size of shards is <25 mm) suitable for further grinding. The resulting pieces were then ground in the planetary ball mill Retsch PM 400 (Haan, Germany), which ensured more refined grinding. Optimal processing parameters were determined for the planetary mill: the grinding duration was 5 min at a rotation speed of 300 rpm.

The resulting crushed mass underwent a fractionation procedure, during which the glass powder was separated into three main fractions: 64–125 microns “125 µm”, 126–250 microns “250 µm”, and 251–500 microns “500 µm”. This fractionation stage is essential for investigating how the particle size fraction affects the final properties of foam glass, structural homogeneity, and energy consumption during production.

An essential step in the production of foam glass is the grinding of perlite, which ensures a uniform particle size necessary for consistent foaming and the formation of a stable material structure. After grinding, the crushed perlite was sieved to isolate a fraction with a particle size of 500 microns, which was identified as the most suitable for producing foam glass with the required properties, by creating more uniform pores distribution with constant pore sizes [19].

The main objective was to investigate how both particle size and particle size distribution (homogeneous versus heterogeneous mixtures) influence foam glass properties such as porosity, mechanical strength, pore structure quality, and process stability. Accordingly, particle size has a critical impact on foaming behaviour, pore formation, and the resulting material characteristics.

Single, homogeneous fractions (125 μm and 250 μm) represent relatively uniform particle sizes, allowing us to study the effects of narrow size distributions on foaming uniformity, structural homogeneity, and mechanical properties. The 125 μm and 250 μm ranges were selected to represent small and medium granulometries commonly encountered in foam glass production, and to observe the effect of increasing particle size.

The mixed heterogeneous fraction (63 + 125 μm) was deliberately prepared as a blend of two smaller size fractions to simulate non-ideal or less controlled particle size distributions as can occur in industrial settings. Including this mixture enables us to examine how heterogeneity affects pore uniformity, structural stability, water absorption, mechanical strength, and the reproducibility of the manufacturing process.

The lower limit fraction (63 μm) was chosen because it represents fine particles near the practical lower limit for effective foaming without excessive densification or poor pore structure. The upper limit fraction (250 μm) was selected because larger particles tend to enhance foaming and strength but may reduce uniformity and control. Studying this range allows us to evaluate a realistic spectrum of granulometries and their effects on foam glass properties.

By including both homogeneous and heterogeneous fractions representative of potential industrial feedstocks, our study offers valuable insights for selecting and optimising raw material preparations to achieve the desired balance between mechanical performance, thermal insulation, water absorption, and process stability.

The foaming agent used in this study (CaCO_3_) was procured in powdered form. To ensure the production of high-quality samples, uniform foaming, and a consistent overall structure of the foam glass, this powder was sieved to obtain a “500 μm” fraction (in the range of 251–500 μm).

### 2.7. Sample Formation and Thermal Treatment

The following components were used in the production of foam glass: cullet glass, crushed perlite, calcium carbonate powder (CaCO_3_), water, and sodium hydroxide (NaOH).

The component proportions were determined as follows: perlite constituted 40% of the total volume of the glass cullet and perlite mixture; CaCO_3_ was added at 2.5 wt% relative to the combined weight of glass and perlite; sodium hydroxide was added at 4 wt% relative to the weight of glass and perlite; water accounted for 10% of the total mass of the mixture (Table 3). The proportions of glass in the mixture “63 + 125 μm” were equal 50/50 by weight.

Initially, the glass, perlite, and CaCO_3_ were mixed in dry form. Subsequently, sodium hydroxide was dissolved in water to form a solution, which was then added to the previously mixed dry components.

**Figure 2:**
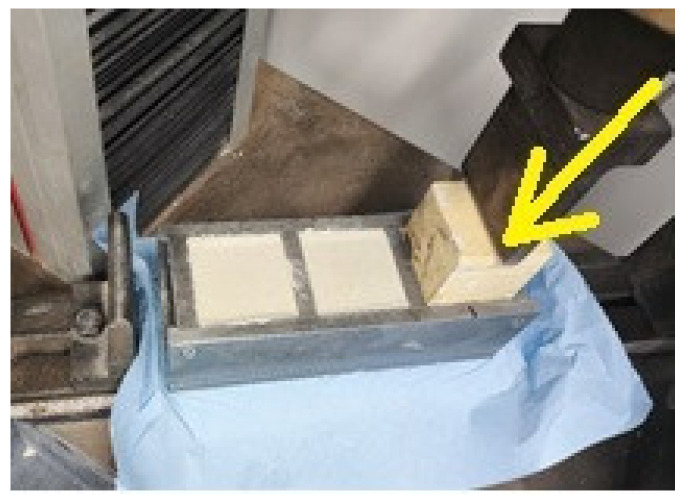
Sample formation with a press.

After preparation and thorough mixing of the components, the foam glass mixture was subjected to a moulding process. The prepared mixture was placed into steel moulds measuring 50 × 50 × 50 mm, enabling the production of samples of uniform size for subsequent testing. To ensure the required mixture density and to prevent sample damage upon removal from the moulds, uniaxial compaction was applied using a press (Figure 2).

The applied load on the sample was 2 kN, which ensured the necessary compaction and improved contact between the particles of the mixture.

Drying of the formed samples prior to high-temperature treatment is a crucial step in the production of foam glass. Immediately after moulding, the samples were placed in a drying chamber and held at a temperature of 68 °C for 24 h. This temperature and drying duration effectively remove excess moisture while preserving the material’s structure and preventing the formation of defects.

There are several important reasons for removing excess moisture from the samples. Firstly, excessive moisture during subsequent high-temperature sintering can disrupt the porous structure of the foam glass. At elevated temperatures, residual water rapidly evaporates, generating excessive internal pressure within the sample, which can lead to damage and microcracks. Such defects adversely affect the strength and thermal insulation properties of foam glass.

The sintering process of dried samples represents the final stage of foam glass production, determining its final physical and mechanical properties. To investigate the effect of temperature on material properties, the samples were sintered with three different sintering schemes with maximum temperatures: 800, 825, and 850 °C. The use of varying sintering temperatures enables the determination of optimal conditions for producing foam glass with the desired properties. Each temperature regime was applied to three samples of the same fraction, ensuring the accuracy of the experiment.

The samples were sintered in multiple stages, with gradual temperature increases to reduce the risk of deformation and improve the homogeneity of the foam glass porosity. In the first stage, the samples were heated to 600 °C at a rate of 10 °C per minute, which allowed for controlled removal of residual moisture and initiation of phase changes in the glass structure. In the second stage, the heating rate was reduced to 3 °C per minute until the samples reached the target maximum temperatures (800, 825, or 850 °C). This regime ensured uniform heating and minimised the likelihood of internal stresses.

**Figure 3:**
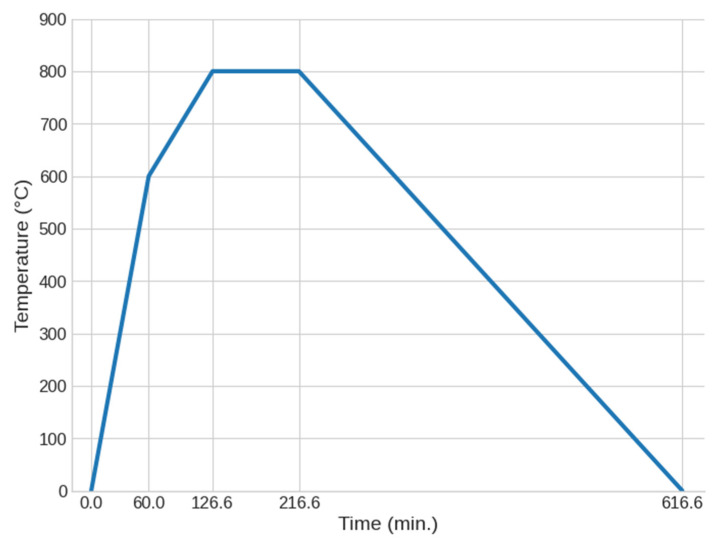
Temperature sintering scheme for 800 °C.

After reaching the maximum temperature, the samples were held at that temperature for 1.5 h, facilitating the stabilisation of the porous structure. The final stage involved cooling the samples at a strictly controlled rate (approximately 2 °C per minute), allowing for a gradual reduction in thermal stress and cracking prevention (Figure 3).

### 2.8. Compressive Strength Testing and Density Definition

To evaluate the mechanical strength characteristics of foam glass, the compressive strength of the samples was tested. Thermally treated samples were prepared (cut) in the form of cubes with dimensions of 40 × 40 × 40 mm, ensuring uniform geometric parameters and minimal differences during testing. The standardised dimensions allowed for the acquisition of reliable and reproducible results.

Before testing, all samples were weighed to determine their mass. Afterwards, based on their volume, the bulk density parameters for each sample group were calculated (relative to the thermal regime and fraction size). This parameter is crucial for further analysis of the relationship between material density and compressive strength.

The test was conducted using a universal testing machine Zwick100, manufacturer ZwickRoell S.a.r.l., produced in Ulm, Germany.

### 2.9. Water Absorption Testing of the Samples

To assess the water absorption capacity of the foam glass, the cubic samples were tested for water absorption. The test samples were prepared in standardised dimensions of 40 × 40 × 40 mm, ensuring consistent testing conditions and minimising potential variations in the results.

The process of determining water absorption included several stages:

- Initial weighing: the dry samples were thoroughly dried and weighed to record their initial mass (m_0_).
- Water Immersion: after weighing, the samples were immersed in a container with water at room temperature. During the moisture absorption process, the samples were sequentially weighed after 1, 2, 3, 24, and 48 h. Weighing continued until the mass of the water-saturated sample stabilised, indicating that maximum water absorption had been reached.

### 2.10. Porosity Testing

To evaluate the porosity of the foam glass, a comprehensive assessment was conducted, including visual characterisation and measurements of the true density of the samples.

As part of the experiment, the foam glass samples were prepared as follows:

- Cutting of plates: initial samples were cut into 3–4 mm thick plates for visual analysis of structure and pore distribution.
- Crushing of samples: for a more detailed study, the samples were crushed to a powdered state to destroy the structure and account for the maximum number of pores.
- Determination of true density: the true density of the samples (*ρ_p_*) was determined using a pycnometer, allowing for precise measurement of the powder material’s volume and eliminating the influence of pores.

The bulk density (*ρ_b_*) for each sample was previously calculated, taking into account the total volume of the material, including the volume of the pores.

### 2.11. Thermal Conductivity Testing

Foam glass thermal conductivity testing was performed using the FOX 600 equipment, manufacturer TA instruments, produced in New Castle, DE, USA, to determine its thermal insulation properties. For the experiment, a large sample with dimensions of 300 × 300 mm and a thickness of 92 mm was fabricated, allowing measurements using specialised equipment.

The foam glass group selected for sample fabrication exhibited an optimal set of properties based on prior testing results. These were samples with a glass particle size of 250 μm and sintering temperatures of 800 °C and 850 °C. The preparation process for the large sample followed the same procedures used in the fabrication of standard cubic samples.

## 3. Results

### 3.1. Compressive Strength and Density

Samples from each batch were tested for compressive strength using the universal testing machine, and their density was calculated by measuring and weighing each sample.

Results of the testing are presented in Table 4.

Reduction in particle size (e.g., to “125 µm”) under identical conditions promotes increased strength (group 6), which may be attributed to improved structural compaction. Group 1 (“500 µm”, 800 °C) exhibits relatively high density (321 kg/m^3^), yet its compressive strength is identical to that of Group 2 (825 °C), which has lower density. This may be due to insufficient sintering at lower temperatures.

To achieve maximum strength with minimal density, preference should be given to group 3 with homogeneous fractions “500 µm” at a sintering temperature of 850 °C. Mixtures with various fractions require further optimisation to improve their structural properties. The sintering temperature plays a crucial role in balancing density and strength, with the 825–850 °C range being the most suitable.

### 3.2. Water Absorption

Water absorption measurements were conducted for each group of samples during the study. Table 5 also presents the water absorption test results for foam glass samples of various fractions at different sintering temperatures. The water absorption rate (percentage of water mass relative to sample mass) is denoted as W, %. The group data are as follows:

Water absorption for the 250 µm fraction is minimal at 800 °C (W = 58%) and increases with rising sintering temperature up to 850 °C. At a lower temperature (800 °C), the material’s structure is likely denser, thereby reducing water uptake. In contrast, at higher temperatures (825–850 °C), a greater number of opened pores form, increasing the material’s ability to absorb water.

Relatively stable water absorption (W = 113–123%) across all sintering temperatures has been obtained for the “250 µm” fraction. The smaller particle size may promote a more uniform pore distribution, making water absorption less temperature-dependent.

The group composed of a “125 + 250 µm” fraction mixture shows significantly higher water absorption than other fractions, peaking at 825 °C (W = 162%). This behaviour is likely due to the non-uniform particle distribution, which results in the formation of larger pores, thereby increasing the material’s capacity to absorb water.

The mixture, consisting of particles “125 + 250 µm”, exhibits the highest water absorption (W = 162%), which limits its use in high-humidity environments but makes it a promising candidate for materials with enhanced thermal insulation properties. A temperature of 850 °C allows for a compromise between mechanical properties and water absorption, especially for the ‘250 µm”, and “500 µm” fractions.

Since the obtained material has a high level of water absorption, it should be protected from access of water during the service life. The construction made from this foam glass should not be applied below the groundwater level. The blocks or panels made from elaborated foam glass should be protected by waterproof mineral plaster or for ventilated facades.

To further reduce water absorption, it is recommended to investigate the effect of additives that alter pore size and structure, but considering existing results and observations, the mixture consisting of particles “125 + 250 µm” fractions showed the worst results, due to unstable porosity (Table 5).

### 3.3. Porosity

The calculated values of true density and porosity for foam glass samples with different particle fractions at various sintering temperatures are presented in Table 5. The data allow for the identification of specific relationships between particle size, sintering temperature, and material porosity.

According to statistical analysis the confidence interval of thermal conductivity for each group is 0.003145 W/mK (500 µm group). 0.00362 W/mK (250 µm group), 0.001413 W/mK (125 + 250 µm group).

“500 µm” fraction: At 800 °C (*ρ_p_*= 2223.9 kg/m^3^, P = 85.6%), the lowest porosity among all samples of this fraction is observed. This indicates a relatively dense structure at the lower sintering temperature. Increasing the temperature to 850 °C (*ρ_p_*= 2191.4 kg/m^3^, P = 87.7%) results in a decrease in density and an increase in porosity, likely due to an increase in the number and size of pores within the material’s structure.

“250 µm” fraction: This fraction exhibits high porosity at all sintering temperatures, reaching a maximum at 850 °C (*ρ_p_*= 2616.6 kg/m^3^). The smaller particle size promotes the formation of a more uniform porous structure, ensuring high but stable porosity regardless of the sintering temperature.

Mixture of particles consisting of fractions “125 + 250 µm”: the highest porosity among all samples is observed, reaching a maximum at 850 °C (*ρ_p_*= 2306.2 kg/m^3^). This can be attributed to the heterogeneity of the mixture, which promotes the formation of larger pores and increases overall porosity.

At a lower temperature (800 °C), samples show reduced porosity, likely due to insufficient sintering of the material.

As the sintering temperature increases, porosity rises, which can be explained by an increase in the number and size of pores in the material structure.

### 3.4. Microscopic Examination

**Figure 4:**
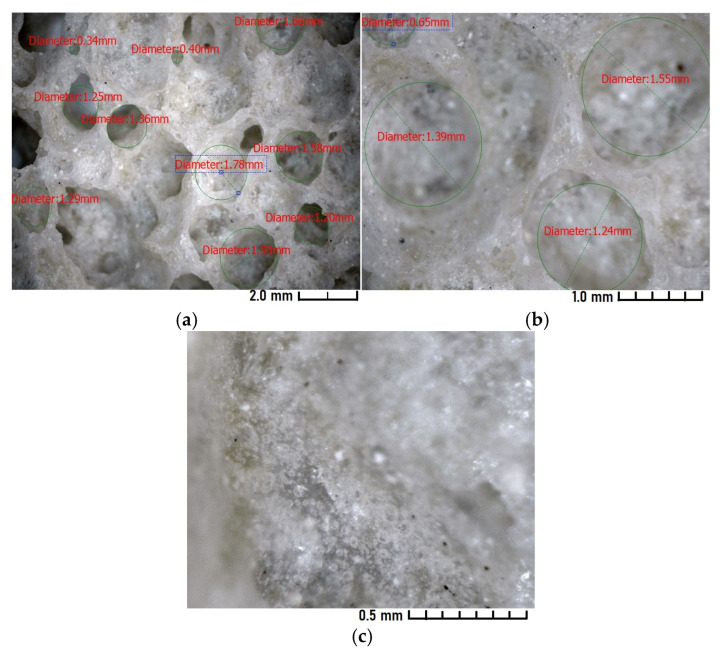
Group 3 (850 °C 500 μm) Microscopic examination: (**a**) magnification 30×; (**b**) magnification 100×; (**c**) magnification 280×.

From Figure 4a, it is seen that pore sizes range from 0.34 mm to 1.78 mm. The structure appears sufficiently uniform, without a pronounced dominance of large pores. The presence of medium-sized pores may indicate an optimal foaming process, suitable for maintaining the mechanical strength of the material.

Well-defined, round-shaped pores (Figure 4b) are visible, indicating a stable foaming process at a temperature of 850 °C.

The fine-pored structure with micropores smaller than 0.02 mm in diameter can be observed in Figure 4c. The material surface appears relatively homogeneous, with uniformly distributed small pores.

**Figure 5:**
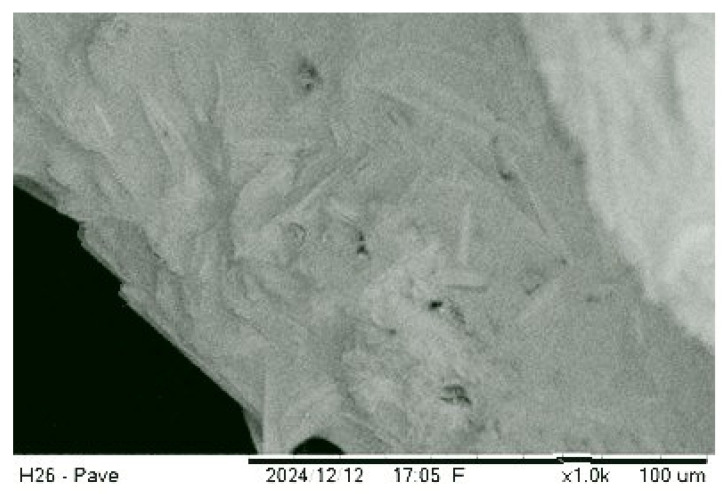
Group 6—850 °C “250 μm” (1000×).

The material surface of the sample from Group 6 exhibits a heterogeneous microstructure (Figure 5). Micropores with diameters of a few micrometres are visible and evenly distributed across the surface. Areas of dense structure are present, indicating effective sintering in specific regions.

The overall appearance of the sample at this magnification confirms the existence of a multi-level structure, where small pores play a critical role in thermal insulation properties. However, further attention is needed to reduce their impact on hydrophobicity. The surface appears sufficiently smooth, indicating a stable foaming process, though individual defects may suggest the need to adjust technological parameters in the manufacturing process.

Sharp edges and linear structures observed in Figure 5 at 1000× magnification may indicate the formation of a crystalline phase in the material. Such structures often arise during partial crystallisation of the glass matrix, which may be a result of specific production conditions.

**Figure 6:**
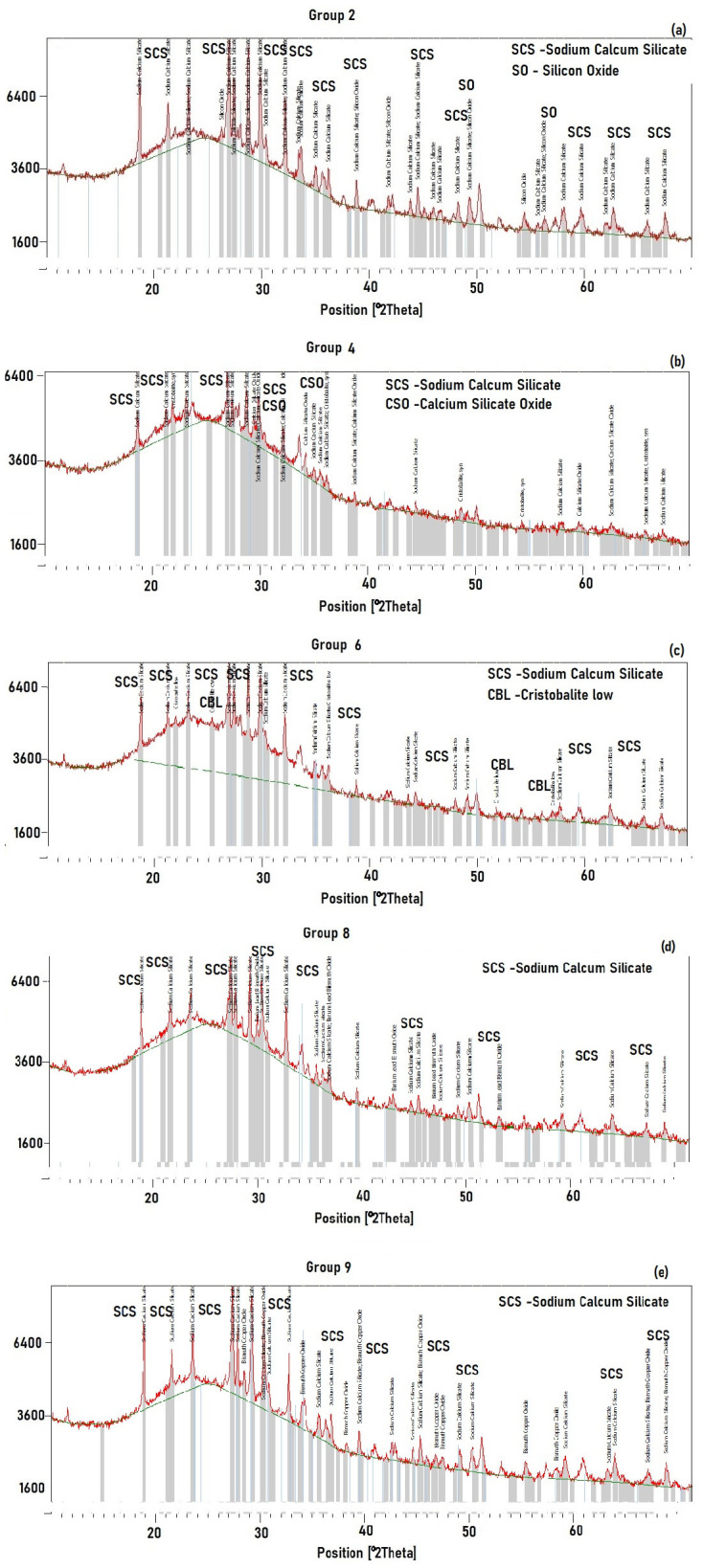
Results of XRD analysis for the composition group 2, 4, 6, 8, and 9. (**a**)—group 2, (**b**) group 4, (**c**)—group 6, (**d**)—group 8 and (**e**)—group 9.

The XRD analysis has been provided for the samples from different groups of considered mixes and sintering temperatures. The results for the compositions groups 2, 4, 6, 8, and 9 are presented in Figure 6a–e correspondingly. The compound Sodium Calcium Silicate predominates in all samples in different proportions.

### 3.5. Thermal Conductivity

The thermal conductivity testing results of foam glass samples are presented in Table 5. The samples Group 1 and Group 3 are recognised as the most promising due to its optimal combination of properties, such as mechanical strength, porosity, density, and visual appearance.

The average thermal conductivity (λ) of the samples under test conditions (T_average = 10.01 °C) was 0.0998 W/mK for the Group 1 and 0.0974 W/mK for the Group 3. These values are low, confirming the good thermal insulation properties of the foam glass. The lowest value of the sample showed a thermal conductivity of 0.0866 W/mK, whereas the highest exhibited a value was 0.0998 W/mK.

In two testing stages (Setpoint 1 and Setpoint 2), the thermal conductivity presented the same stable values (λ = 0.0974 W/mK for Group 3), which indicates the material’s reliability and the stability of its properties.

Comparison of the materials listed in Table 5 and Table 6 reveals that they have different intended applications and effectiveness in thermal insulation and structural functions. Analysis of thermal conductivity (λ), density (ρ), and compressive strength (σ) highlights the key properties of each material.

By comparison with traditional insulation materials, it can be concluded that extruded polystyrene foam (λ = 0.029 W/mK) and polyurethane foam (λ = 0.029–0.041 W/mK) exhibit the lowest thermal conductivity values, making them the most efficient thermal insulation materials. At the same time, foam glass, including the Group 3 (λ = 0.0974 W/mK), exhibits higher thermal conductivity while retaining competitiveness due to additional properties such as durability, longevity, and non-combustibility.

The samples 2, 4, 6, 8, and 9 were chosen for XRD analysis as representative points covering different particle size fractions (125 μm and 63 + 125 μm mixtures) and sintering temperatures, specifically targeting compositions and conditions exhibiting distinct mechanical and physical properties. This selection allows for capturing the phase evolution influenced by both particle size distribution and sintering temperature, thereby providing meaningful insights into structure–property relationships.

The XRD patterns indicate a predominantly amorphous glass matrix with varying degrees of crystallinity depending on processing conditions. While qualitative peaks confirm the presence of crystalline phases, quantitative phase analysis via Rietveld refinement or other methods was not performed in this study.

The XRD patterns for groups 2, 4, 6, 8, and 9 (Figure 6) reveal the primary crystalline phases present in each sample, mainly centred around sodium calcium silicate (SCS), along with some secondary phases such as silicon oxide and cristobalite. Dominant Phase: The majority of the patterns show intense peaks labelled as SCS, indicating that sodium calcium silicate is the predominant phase in these group compositions. Secondary Phases: Peaks corresponding to silicon oxide (SO) and cristobalite low (CBL) are also present in some groups, suggesting partial crystallisation of silica-based phases, possibly due to reaction or thermal treatment conditions. In Groups 2 and 4, the SCS peaks are especially prominent, while minor peaks for silicon oxide or cristobalite suggest possible incomplete reaction or phase separation. Groups 6, 8, and 9 show some additional labelled peaks (CSO—calcium silicate oxide, CBL—cristobalite low), further supporting the coexistence of secondary silicate phases. Peak assignments indicate crystallinity and the repeated presence of sodium calcium silicate across all groups, which confirms successful formation of the target compound through synthesis or processing. The appearance of cristobalite in certain groups points to high-temperature phase transformations of the silica component. Minor impurity peaks or phase overlaps suggest small compositional or structural variations between groups, possibly attributable to differences in synthesis temperature, precursors, or reaction times.

The predominance of SCS implies that the composite is primarily composed of robust silicate crystals, likely imparting desirable chemical and thermal stability. Secondary phases (silicon oxide, cristobalite) may affect mechanical or thermal properties, potentially acting as weak spots or alternatively reinforcing the silicate matrix, depending on their distribution and morphology. All groups share the core feature of strong SCS peaks but differ in the presence and intensity of secondary phases. This points to an effective synthetic strategy for silicate-based ceramics/composites, with fine-tuning of thermal or chemical parameters influencing phase purity and crystalline structure.

The detected crystalline phases mainly include [list the identified phases if specified, e.g., cristobalite, quartz, etc.]. Their relative proportions appear to vary with sintering temperature and composition heterogeneity. However, due to the semi-quantitative nature of the current analysis and overlapping peaks, precise quantification was not possible.

Preliminary observations from the XRD results suggest that compositions with more homogeneous particle sizes (e.g., 125 μm fraction) show fewer and weaker crystalline peaks compared to heterogeneous mixtures (63 + 125 μm), which exhibit sharper peaks indicative of increased crystallinity, likely due to enhanced nucleation sites. Additionally, higher sintering temperatures promote partial crystallisation, as shown by the emergence or intensification of specific peaks. These phase changes can correlate with the variations in mechanical strength and stability seen in the samples.

## 4. Discussion

### 4.1. Mechanical Properties

Compressive strength improves significantly with the increase in particle size of the used fraction. Composition consisting of particles from the “500 μm” fraction provides the highest compressive strength (2.17 MPa) due to its ability to form a homogeneous and structurally stable matrix (Table 5). This trend suggests that coarser fractions positively affect foaming uniformity and reduce the formation of defects. In contrast, the composite fraction consisting of particles “125 + 250 μm” exhibits significantly lower strength, possibly due to particle heterogeneity, which compromises its structural integrity.

Firing temperature is another critical factor influencing mechanical properties. In most groups, the optimal temperature is 850 °C, at which the highest strength is achieved without significantly reducing porosity. However, it has been observed that continuing to increase the temperature risks excessive melting, which may degrade the porous structure and its thermal insulation properties. While perlite itself is stable at high temperatures and does not directly lower the softening point of the glass, its inclusion allows for a reduction in the overall glass content and promotes the formation of a more uniform pore structure. Furthermore, the addition of sodium hydroxide as an alkaline component plays the primary role in lowering the melting/foaming temperature. Therefore, the combination of using perlite along with sodium hydroxide and fine-tuning particle sizes achieves the targeted reduction in foaming temperature while maintaining or improving the material properties.

**Figure 7:**
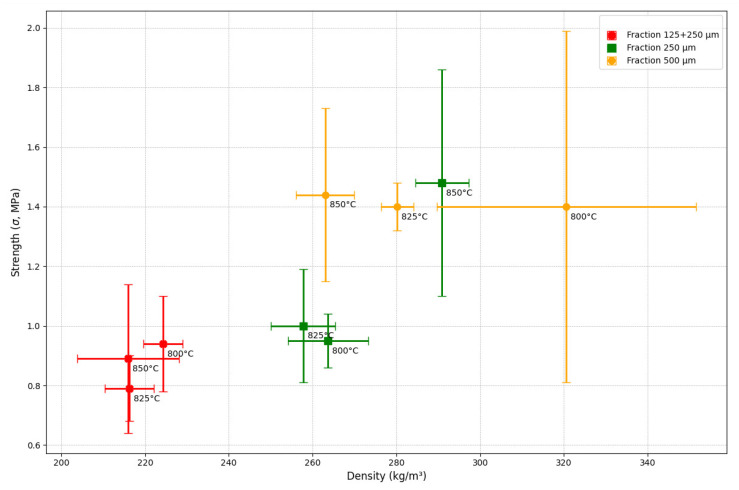
Strength-density ratio for foam glass samples.

As shown in Figure 7, all samples exhibit a common trend: with decreasing density, compressive strength either decreases or increases up to a certain threshold. This suggests that density is not the sole determinant of strength; the quality of the pore structure plays a primary role.

Samples with a “500 µm” glass fraction demonstrate the best balance between density and strength, particularly at a sintering temperature of 850 °C (Group 3). This makes them most suitable for applications where high strength is required (Table 4).

Samples with lower density (216 kg/m^3^, groups 8 and 9) exhibit limited strength values (up to 0.93 MPa). This suggests that such particle mixtures are less effective in forming durable structures compared to homogeneous fractions. However, they may be suitable for producing materials with high thermal insulation properties.

For the “500 µm” fraction, the horizontal error bars are very large (high standard deviation in density), but the vertical error bars are very small (low standard deviation in strength). This means that when using this fraction, the resulting strength is very predictable, but the density is very unstable and difficult to replicate.

For the “250 µm” fraction, the vertical error bars are the largest. This means that the strength measurements for this fraction were the most variable.

For the “125 + 250 µm” fraction, the error bars are the smallest in both directions. This means that although this fraction produces a weaker and less dense material, the process itself is the most stable, and the results are the most easily reproducible.

The “500 µm” fraction yields the strongest material, but the manufacturing process is unstable (it is difficult to control the density). In contrast, the “125 + 250 µm” fraction yields a weaker material, but the process is very stable, and the results are predictable. This information is critically important when choosing a material and manufacturing method for real-world applications.

A temperature of 825 °C is suitable for most samples, providing an acceptable level of density and strength. However, to achieve maximum strength, as observed in the case of Group 3, a temperature of 850 °C is preferable.

### 4.2. Influence of Porosity on Mechanical and Physical Properties

**Figure 8:**
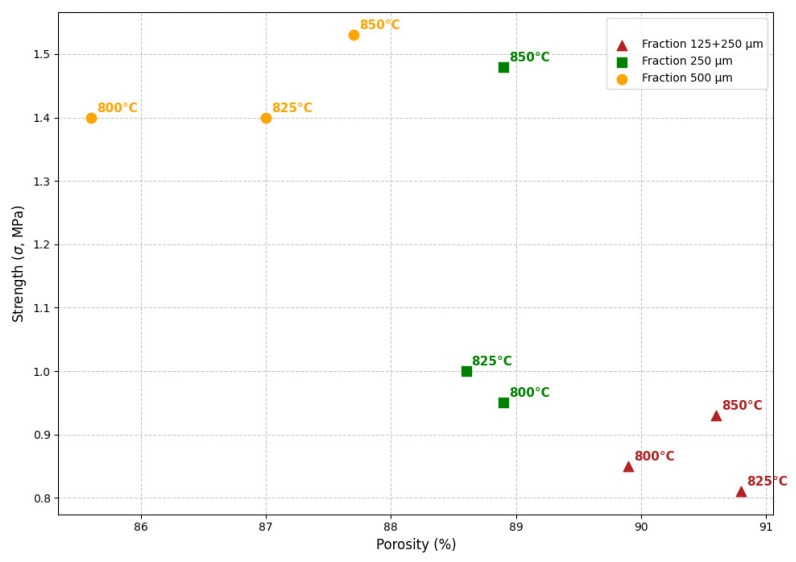
Strength-porosity relationship of foam glass samples.

The relationship between porosity and mechanical properties is complex. It was revealed that for compositions consisting of the “500 μm” fraction, despite an increase in porosity (from 85.6% to 87.7%), compressive strength increases, indicating an improvement in structural density (Figure 8). In contrast, for the heterogeneous composition with particles fraction “125 + 250 μm”, an increase in porosity results in a pronounced decrease in compressive strength, indicating an ineffective pore arrangement and structural defects.

**Figure 9:**
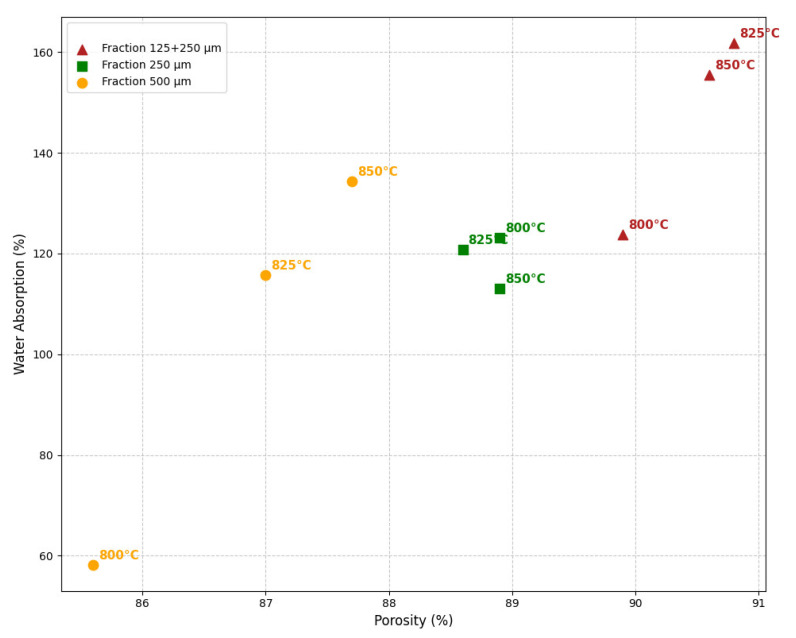
Water absorption-porosity relationship of foam glass samples.

Similarly, water absorption rates correlate with porosity—higher porosity results in higher water absorption (Figure 9). This effect is particularly pronounced in the case of the heterogeneous fraction (increase from 123.8% to 161.8%), indicating the presence of a large volume of open pores. In contrast, the composition made from “250 μm” fraction particles exhibits much more stable water absorption values, despite slight fluctuations in porosity, indicating a more uniform structure.

**Figure 10:**
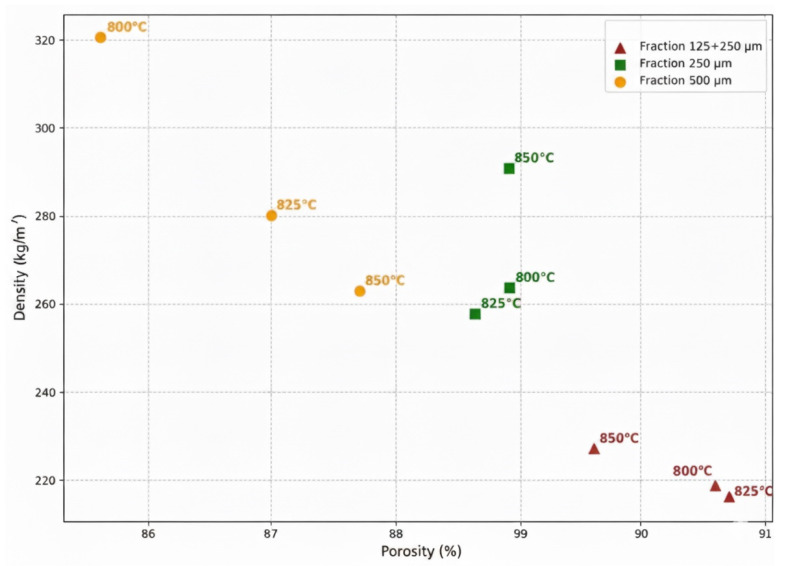
Density-porosity relationship of foam glass samples.

An increase in porosity typically results in a decrease in density (Figure 10). This trend is most pronounced in the “500 μm” fraction, where the density decreases from 321 to 263 kg/m^3^, indicating an effective foaming process and the development of open porosity. Lower density values in heterogeneous fractions indicate structurally less stable material.

### 4.3. Quality of Pore Structure and Importance of Manufacturing Parameters

The quality of the pore structure is significantly dependent on the homogeneity of the fractions and the parameters of the thermal process [22,23,24]. In groups where homogeneous fractions were used, such as “250 μm” and “500 μm”, the porosity is uniform, and the structure is stable. In contrast, samples with composite fractions demonstrate uneven pore distribution and weak mechanical stability.

The heating rate used in the study (3 °C/min) proved sufficiently effective for homogeneous fractions; however, even more precise control, especially for smaller fractions, may improve results. The use of sodium hydroxide (NaOH) has shown significant importance as a flux stabiliser and foaming catalyst. Its optimal concentration should be determined in future studies.

### 4.4. Influence of Crystalline Phase

The formation of a crystalline phase is one of the most critical factors that can negatively affect the properties of foam glass. The presence of crystalline structures promotes increased thermal conductivity while also creating zones of mechanical brittleness within the material. Therefore, strict temperature control (within the 825–850 °C range), optimal selection of holding time, and uniform cooling—particularly in the critical 600–800 °C interval—are required [2]. Additionally, sodium hydroxide and other additives, such as aluminium or boron compounds, may effectively reduce the formation of the crystalline phase.

### 4.5. Comparative Characteristics of Foam Glass Materials with Standards

The appropriate requirements for foam glass materials utilised in building and insulation applications, including compressive strength and thermal conductivity parameters specified in ASTM C552 [25] and EN 13055-1 [26]. The comprehensive comparative data illustrating (Table 6) how obtained materials meet these norms, substantiated by relevant references, is provided.

## 5. Conclusions

Based on the conducted study, the primary objectives regarding the optimisation of porous glass composition and production technology were successfully achieved. The results confirmed that homogeneous particle size fractions (specifically “250 μm” and “500 μm”) ensured significantly better physical and mechanical properties compared to mixed particle fractions (“125 + 250 μm”), which led to structural non-uniformity and reduced performance. Compression strength according to groups is next: Groups 1–3 (“500 μm”) = 1.63 MPa (190%); Groups 4–6 (“250 μm”) = 1.14 MPa (133%); Groups 7–9 (“125 + 250 μm”) = 0.86 MPa (100%). The optimal foaming temperature was identified in the range of 825–850 °C, ensuring a favourable balance between porosity, compressive strength, and thermal stability. Increasing the particle size improved compressive strength, confirming the importance of proper raw material preparation.

Microscopic and physical analyses revealed that the produced foamed glass predominantly exhibited open porosity, which increased water absorption and affected thermal insulation. To enhance the material, further work is needed to promote the formation of closed pores through the controlled use of additives and the regulation of firing atmosphere. The presence of crystalline phases was also detected, which may reduce mechanical integrity and thermal insulation. This highlights the importance of strict thermal control and the use of stabilising additives to maintain an amorphous structure.

The developed preparation method, which includes controlled mixing, moisture content management, and the use of vibration during forming, significantly improves structural uniformity. Additionally, the obtained samples demonstrated comparable thermal conductivity to industrial foamed glass products while achieving higher compressive strength, indicating their suitability for practical applications. The use of recycled materials such as glass waste and perlite, combined with a simplified processing approach, confirms the method’s economic and environmental feasibility. Overall, the study validates the potential of producing different types of porous glass for construction and insulation applications.

## Figures and Tables

**Table 1:** Glass chemical composition.

Oxides	SiO_2_	Na_2_O	CaO	MgO	Al_2_O_3_	Fe_2_O_3_
Part, %	70–75	12–15	10–12	1–3	1–3	<1

**Table 2:** Perlite chemical composition.

Oxides	SiO_2_	Al_2_O_3_	Fe_2_O_3_	TiO_2_	CaO	MgO	K_2_O	Na_2_O	LOI
Part, %	72.4 ± 1.2	13.3 ± 0.6	1.2 ± 0.2	0.2 ± 0.1	1.1 ± 0.2	0.3 ± 0.1	4.0 ± 0.4	4.0 ± 0.3	3.4 ± 0.8

**Table 3:** Proportions of materials in each group and sintering temperatures.

Group	Temp., °C	Glass, %	Perlite, %	NaOH, %	CaCO_3_, %	Water, %
1	800	66.04 “500 μm”	19.32	3.41	2.15	9.08
2	825	66.04 “500 μm”	19.32	3.41	2.15	9.08
3	850	66.04 “500 μm”	19.32	3.41	2.15	9.08
4	800	66.04 “250 μm”	19.32	3.41	2.13	9.10
5	825	66.04 “250 μm”	19.32	3.41	2.13	9.10
6	850	66.04 “250 μm”	19.32	3.41	2.13	9.10
7	800	66.04 “125 + 250 μm”	19.32	3.41	2.13	9.10
8	825	66.04 “125 + 250 μm”	19.32	3.41	2.13	9.10
9	850	66.04 “125 + 250 μm”	19.32	3.41	2.13	9.10

**Table 4:** Compressive strength and density results.

Group	Size, μm	Temp., °C	Density, kg/m^3^	σ, MPa	STDEV, Density	STDEV, Strength
1	“500 μm”	800	321	1.40	30.93	0.59
2		825	280	1.40	3.87	0.08
3		850	269	1.53	6.98	0.29
4	“250 μm”	800	264	0.95	9.57	0.09
5		825	258	1.00	7.72	0.19
6		850	291	1.48	6.33	0.38
7	“125 + 250 μm”	800	224	0.85	4.68	0.16
8		825	216	0.81	5.83	0.11
9		850	216	0.93	12.13	0.25

**Table 5:** Samples porosity, true density and thermal conductivity results.

Group	Size, μm	Temp., °C	W, %	True Density, kg/m^3^	Porosity, %	Thermal Conductivity, W/mK	STDEV, Thermal Conductivity	STDEV, True Density	STDEV, Porosity
1	“500 μm”	800	58	2223.9	85.6	0.0998	0.0016	214.575	1.39060
2		825	116	2156.6	87.0	0.0979	0.0008	29.772	0.17936
3		850	134	2191.4	87.7	0.0974	0.0015	56.763	0.31834
4	“250 μm”	800	123	2375.7	88.9	0.0956	0.0014	86.250	0.40294
5		825	121	2267.7	88.6	0.0963	0.0011	67.969	0.34062
6		850	113	2616.6	88.9	0.0984	0.0009	56.902	0.24174
7	“125 + 250 μm”	800	124	2217.3	89.9	0.0877	0.0017	46.276	0.21115
8		825	162	2349.6	90.8	0.0866	0.0006	63.304	0.24792
9		850	155	2306.2	90.6	0.0869	0.0016	129.536	0.52589

**Table 6:** Comparison of the properties of the obtained materials with the data in the standards for foam glass.

Property	Measured Value (Current Study)	Typical Standard Value ASTM C552/EN 13055-1	Comment on Compliance
Compressive Strength	Up to 3.5 MPa (Group 3, 850 °C)	≥1.5 MPa for structural foamed glass (min.)	Exceeds minimum requirement
Porosity	85.6–87.7% (open porosity predominant)	Typically 70–90% porosity in insulating foamed glass	Within typical range
Thermal Conductivity (λ)	0.0866–0.0998 W/m·K (average ~0.0974 W/m·K)	≤0.10 W/m·K for insulation materials	Slightly better than standard
Density	263 – 321 kg/m^3^ (depending on fraction)	Generally <500 kg/m^3^ for insulating foam glass	Within requirements
Structural Stability	Stable strength with “250–500 μm” particle fraction; process reproducibility improved by controlled mixing and moisture management	Consistent quality expected for practical applications	Corresponds to this sample selection.

## Data Availability

The original contributions presented in the study are included in the article. Further inquiries can be directed to the corresponding author.
